# Characterization of a *Monanema* nematode in *Ixodes scapularis*

**DOI:** 10.1186/s13071-020-04228-6

**Published:** 2020-07-24

**Authors:** Rafal Tokarz, Teresa Tagliafierro, W. Ian Lipkin, Adriana R. Marques

**Affiliations:** 1 Center for Infection and Immunity, Mailman School of Public Health, Columbia University, New York, NY USA; 2 Laboratory of Clinical Immunology and Microbiology, National Institute of Allergy and Infectious Diseases, National Institutes of Health, Bethesda, MD USA

**Keywords:** *Ixodes scapularis*, Nematode, *Filaria*, *Monanema*, Lyme disease

## Abstract

**Background:**

Metagenomic studies have revealed the presence of a filarial nematode in *Ixodes scapularis*. The phylogeny of this agent, and its potential for human infection, are unknown.

**Methods:**

We used existing metagenomic data from *I. scapularis* to determine the phylogeny of this tick-associated nematode and employed quantitative PCR to determine if the presence of this agent had an effect on the burden of *Borrelia burgdorferi*. We also developed a Luciferase Immunoprecipitation System assay using the Av33 antigen as a target to investigate the presence of antibodies against this nematode in 128 serum specimens from patients with Lyme disease and babesiosis. To demonstrate assay utility, we used 15 sera from patients with onchocerciasis as controls.

**Results:**

We show that this agent is a new species in the genus *Monanema* and its presence in vector ticks does not impact the burden of *B. burgdorferi*. We did not detect IgG antibodies to this agent in 127 of 128 sera from patients with Lyme disease or babesiosis. One sample had reactivity above the threshold, but at the low-level equivalent to the least reactive onchocerciasis sera. This low positive signal could be a result of cross-reacting antibodies, antibodies from a previous infection with a filarial nematode, or, less likely, a exposure to the *Ixodes scapularis-*associated nematode.

**Conclusions:**

We found no evidence that this nematode contributes to the spectrum of human tick-borne infections.
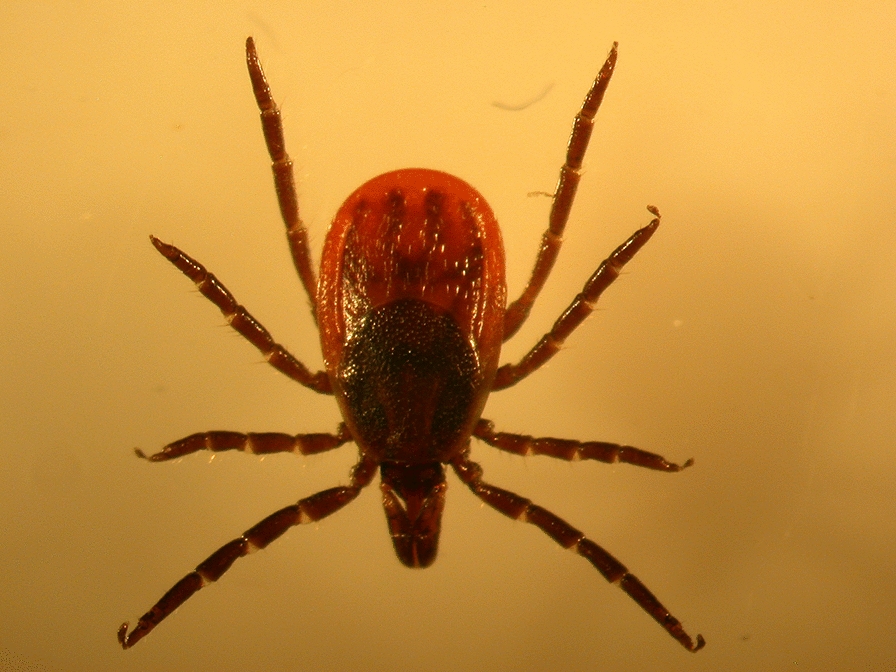

## Background

The blacklegged tick, *Ixodes scapularis*, is arguably the most clinically significant tick species in the USA. In 1982, Burgdorfer and colleagues identified *Ixodes scapularis* as a vector of *Borrelia burgdorferi*, the agent of Lyme disease [1]. Along with this discovery, the group also noted the presence of motile microfilariae in approximately 1% of adult ticks they examined [2]. After this initial report, the identity of this agent remained obscure for the next three decades. In 2014, *12S* rRNA gene sequences homologous to sequences from filarial nematodes were detected in *I. scapularis* ticks in Connecticut [3]. Subsequent metagenomic analyses of *I. scapularis* from Wisconsin and our study from New York and Connecticut also reported Filarioidea sequences [4, 5]. Because filarial nematodes are implicated in veterinary and human disease and there is a high degree of human contact with *I. scapularis,* we sought to genetically characterize this nematode (tentatively named *Ixodes scapularis-*associated nematode, or ISN) and determine the level of human exposure.

## Methods

We used metagenomics data from Tokarz et al. [5] to obtain Filarioidea sequences used in this study. All alignments were performed in Geneious version 10.2 software. For PCR analyses, we developed quantitative TaqMan PCR assays using the primers (forward: 5′-AGC TGA AGA GCT TGG AAT GC-3′; reverse: 5′-GGT TTG CTC CAA CAT GAA CTC-3′) and probe Fam (CAC CAG CAT CAC TTT CAG GGT CTC AA Tamra) for the *B. burgdorferi flab* gene, and (forward: 5′-AGT GCT GGA GGC GAA CGT AA-3′; reverse: 5′-GAC AAC GCA TCC GGC AGT TC-3′) and probe Vic (TCG CTG GAT TCA ACG CTG CTG GA Tamra) for the ISN *av33* gene. For serological tests, we developed a Luciferase Immunoprecipitation Systems (LIPS) assay [6] for the ISN AV33 antigen. We cloned a DNA fragment with the *I. scapularis* AV33 mRNA sequence into pREN vector and expressed the luciferase-AV33 fusion protein in Cos-1 cells. Serum IgG antibody binding to AV33 was measured by fluorescence intensity on a illuminometer. Background was determined by testing negative controls consisting of buffer (*n* = 24) or deidentified sera negative for tick-borne diseases (*n* = 5). Positivity threshold was established by calculating the mean plus 5 standard deviations.

De-identified sera collected from patient with Lyme disease and onchocerciasis were obtained under clinical protocols (ClinicalTrials.gov Identifier NCT00028080, NCT00001539 and NCT00001230) approved by the institutional review board of the National Institute of Allergy and Infectious Diseases, and all participants signed informed consent. De-identified samples from patients with babesiosis and sera from *Peromyscus leucopus* were kindly provided by Dr Azad Gucwa and Dr. Sam Telford, respectively.

## Results

To clarify the phylogenetic placement of ISN, we mined our metagenomic data and assembled sequences of informative genes for taxonomic determination. Homology searches of the *18S* rRNA gene (GenBank: MK868471) revealed 100% and 98.9% matches to sequences KP760148 and KP760149 that correspond to a 665-nucleotide fragment of *18S* rRNA from *Monanema martini*.

**Fig. 1:**
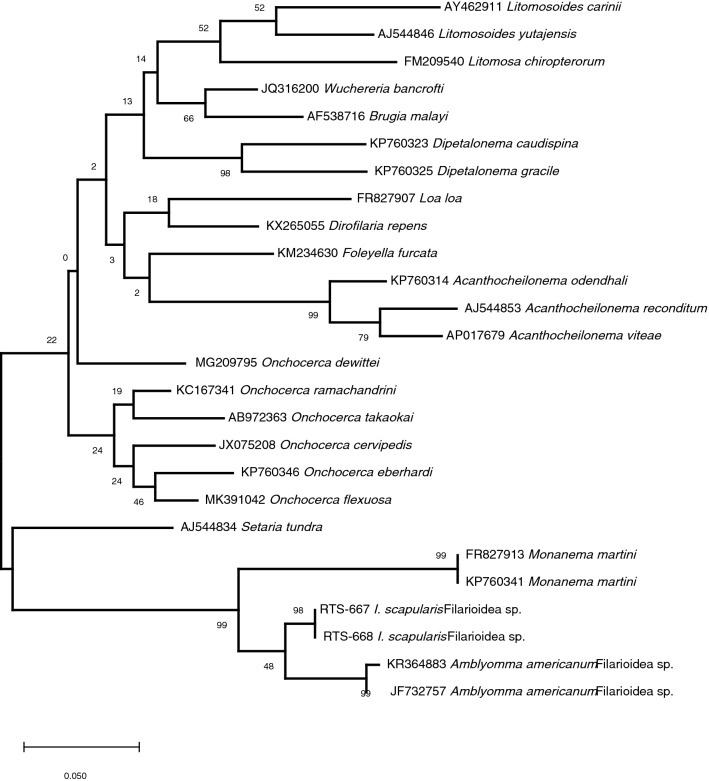
Phylogeny of the *Ixodes scapularis-*associated nematode (ISN). The phylogenetic tree was generated using a 455 nt fragment of the ISN *12S* rRNA gene. RTS-667 and RTS-668 represent sequences obtained in this study

*Monanema martini* is a filarial nematode identified in Africa that infects at least two species of African rodents, the typical striped grass mouse (*Lemniscomys striatus*), and the African grass rat (*Arvicanthis niloticus*) with *Hyalomma* and *Rhipicephalus* ticks implicated as vectors [7, 8]. We used consensus PCR to obtain sequences of a 455 nt fragment of the ISN *12S* rRNA gene that is often used for phylogenetic assignment of Filarioidea. The ISN sequences were homologous to the sequences reported from *I. scapularis* from Connecticut in 2014, and were also clustered with nematode sequences previously identified in *Amblyomma americanum* ticks in Virginia and Maryland (92%) and *M. martini* (88%) [9] (Fig. 1). We conclude that the nematodes in *A. americanum* and *I. scapularis* along with *M. martini* form a distinct phylogenetic cluster within Filarioidea and all three are likely distinct species within genus *Monanema.* Next, we wanted to determine if ISN is also present in *A. americanum*. We tested 50 adult *A. americanum* collected on Long Island, NY and New York City, and all were negative for ISN. Conversely, 14% (*n* = 27) of adult *I. scapularis* were positive for ISN from these areas. The majority (*n* = 17) of ISN-infected *I. scapularis* were also positive for *B. burgdorferi*. To determine if the presence of ISN affects the burden of *B. burgdorferi*, we used a qPCR assay targeting the *B. burgdorferi* single copy *flaB* gene. We observed no significant variation in *B. burgdorferi* bacterial load in ticks with or without ISN.

**Fig. 2:**
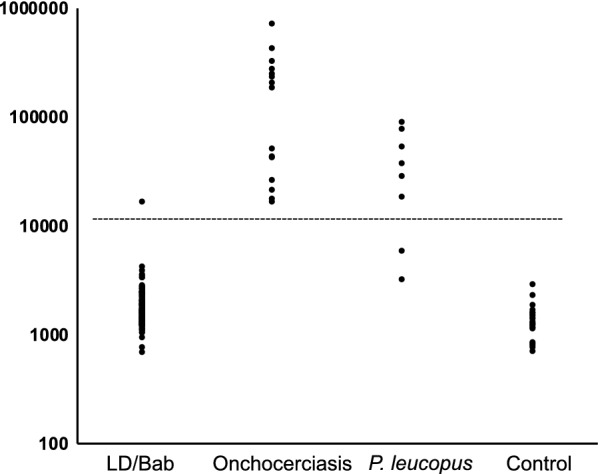
Detection of anti-Filarioidea IgG antibodies by Luciferase Immunoprecipitation System assay. The antibody titers shown in light units are plotted on the y-axis in log-scale. Shown are the results of the sera from Lyme disease or babesiosis patients (*n* = 128), patients with onchocerciasis (*n* = 15), sera from *Peromyscus leucopus* (*n* = 8) and negative controls (*n* = 29). The dashed line represents the diagnostic cutoff and is derived from the mean antibody titer of negative controls plus 5 standard deviations

Since *Monanema* species in Africa exist in an enzootic cycle between ticks and mammals, it is plausible ISN could infect vertebrates, and that humans would occasionally be exposed to this agent. We employed a serological approach, using a LIPS assay, to search for evidence of human exposure to ISN [6]. Based on published serological studies for filariasis, we selected the AV33 antigen as an appropriate antigenic target and assembled the complete nucleotide sequence of the *av33* gene using our metagenomic data and PCR (GenBank: MN756545). The complete open reading frame consists of 4 introns and 5 exons, with the coding sequence of 699 nt that results in a 232 amino acid (aa) putative protein. The closest sequence in GenBank to ISN *av33* belongs to *Acanthocoilonema vitae* (83% nt and 76% aa identity). *Monanema* sequences were not present on GenBank (as of April 2020). The coding sequence of ISN Av33 was amplified and cloned into a pREN vector and expressed as a fusion protein with *Renilla* luciferase. Since Av33 shares substantial homology within all Filarioidea to establish thresholds for reactivity we used 15 de-identified sera from patients with onchocerciasis with the assumption that antibodies to non-ISN filarial Av33 would cross-react with ISN Av33. This approach was suitable, as all 15 sera from patients with onchocerciasis were positive by LIPS. Next, we examined 128 de-identified sera collected during acute disease or at convalescence from individuals diagnosed with either Lyme disease or babesiosis. From these 128 samples, 127 were negative (Fig. 2). One sample had reactivity above the threshold, but at the low-level equivalent to the least reactive onchocerciasis sera. Conversely, when we tested 8 sera from *Peromyscus leucopus* mice that often serve as the hosts for immature stages of *I. scapularis*, 6 sera tested positive.

## Discussion

We determined that ISN along with the nematode found in *A. americanum* represent the first species of the genus *Monanema* uncovered outside of Africa. Recent insights into the *I. scapularis* microbiome have demonstrated that the prevalence of ISN is comparable to the prevalence of some *I. scapularis*-vectored pathogens [5, 10]. In addition, its presence in ticks from the Mid-West and eastern USA suggest that ISN may have a geographical distribution throughout the range of *I. scapularis*. Nonetheless, we were not able to demonstrate antibodies to filarial nematodes in patients with previous exposure to *I. scapularis* bites in all but one sample which yielded a low positive signal with LIPS. While this low positive signal could be a result of ISN exposure, it could also be caused by antibodies from a previous infection with a non-ISN filarial nematode or other cross-reacting antibodies. In contrast, we also tested sera from *P. leucopus*, which, similar to other *I. scapularis*-transmitted agents, could presumably serve as a reservoir host for ISN. Six of the eight sera tested were positive, but similarly, we cannot exclude that this reactivity was due to other nematode infections.

## Conclusions

Our data show that human exposure to ISN is negligible and this agent is highly unlikely to contribute to the spectrum of human tick-borne infections.

## Data Availability

The datasets analyzed in this study are available under the following link: https://www.ncbi.nlm.nih.gov/bioproject/PRJNA589083.

## Abbreviations

LIPS: Luciferase Immunoprecipitation Systems assay
ISN: *Ixodes scapularis-*associated nematode
